# Osteoporosis pathogenesis and treatment: existing and emerging avenues

**DOI:** 10.1186/s11658-022-00371-3

**Published:** 2022-09-04

**Authors:** Bo Liang, George Burley, Shu Lin, Yan-Chuan Shi

**Affiliations:** 1 Department of Endocrinology and Metabolism, The Second Affiliated Hospital of Fujian Medical University, Quanzhou, China; 2 Neuroendocrinology Group, Garvan Institute of Medical Research, 384 Victoria Street, Darlinghurst, Sydney, NSW 2010 Australia; 3 Centre of Neurological and Metabolic Research, The Second Affiliated Hospital of Fujian Medical University, No.34 North Zhongshan Road, Quanzhou, 362000 Fujian Province China; 4 St Vincent’s Clinical School, Faculty of Medicine, UNSW Sydney, Sydney, Australia

**Keywords:** Osteoporosis, Pathogenesis, Bone remodeling, Bone formation, Bone resorption, MicroRNA-based therapy, Stem cell therapy

## Abstract

Osteoporotic fractures lead to increased disability and mortality in the elderly population. With the rapid increase in the aging population around the globe, more effective treatments for osteoporosis and osteoporotic fractures are urgently required. The underlying molecular mechanisms of osteoporosis are believed to be due to the increased activity of osteoclasts, decreased activity of osteoblasts, or both, which leads to an imbalance in the bone remodeling process with accelerated bone resorption and attenuated bone formation. Currently, the available clinical treatments for osteoporosis have mostly focused on factors influencing bone remodeling; however, they have their own limitations and side effects. Recently, cytokine immunotherapy, gene therapy, and stem cell therapy have become new approaches for the treatment of various diseases. This article reviews the latest research on bone remodeling mechanisms, as well as how this underpins current and potential novel treatments for osteoporosis.

## Introduction

In the face of an increasingly aging population, osteoporosis (OP) is becoming one of the most common diseases worldwide. At the time of the last US Census in 2010, the overall prevalence of osteoporosis in adults aged 50 years and older was approximately 10.2 million. The prevalence was significantly higher in women (16.5%) than in men (5.1%) [1]. Osteoporosis is characterized by deteriorated bone strength and a subsequent increase in fracture risk [2]. Osteoporotic fractures, or fragility fractures, are responsible for significant reductions in quality of life, as well as increased social and economic burdens at an individual and population level. This is particularly true for hip fractures; within a year of sustaining a hip fracture for those aged over 50 years, approximately 20% of patients will be dead, and nearly 50% of patients will be disabled [3].

The clinical diagnosis of osteoporosis is based mainly on bone mineral density (BMD), measured using dual-energy X-ray absorptiometry (DEXA), and/or the occurrence of fragility fractures [2]. The fracture risk prediction tool (FRAX), recommended by the World Health Organization (WHO), can be used to evaluate the incidence of osteoporotic fractures. This prediction tool includes major risk factors for osteoporotic fracture: age, sex, body mass index (BMI), fracture history, smoking, glucocorticoid medication history, rheumatoid arthritis, diseases that can cause secondary osteoporosis, and BMD [4]. At present, the treatment of osteoporosis is based on its pathogenesis, which is studied at different stages of disease development.

Bone consists of dense outer cortical bone and spongy inner cancellous bone, both having distinct properties that work together to maintain bone strength. They are made up of cells, including osteocytes, osteoclasts, osteoblasts and stem cells, and bone matrix, which is composed of calcium, phosphorus, inorganic salts and bone collagen. Osteoclasts resorb bone, whereas osteoblasts form new bone. The antagonistic actions of these two cell types occur constantly in the body in order to maintain bone health and structural integrity of the skeleton. This process is termed bone remodeling or bone turnover [5]. Any factors that decrease the activity of osteoblasts and/or increase the activity of osteoclasts will result in greater bone resorption than bone formation. This imbalance in bone remodeling also induces the destruction of bone microstructure, especially the structural destruction of cancellous bone, which leads to a decrease in bone strength and subsequent fragility fractures.

By exploring the underlying molecular mechanisms of imbalances in bone remodeling, novel osteoporosis treatments have been developed. Bisphosphonates, acting to inhibit bone resorption, are one such example, whose clinical application has brought revolutionary changes to osteoporosis treatment [6]. Another example is denosumab, a monoclonal antibody targeting the nuclear factor kappa B (NF-κB) ligand activated receptor (RANKL), serving to slow bone breakdown [7]. Its clinical application in recent years displays the successful application of cytokine immunotherapy in osteoporosis treatment [8]. However, both bisphosphonates and denosumab still have limitations and side effects, such as mandibular osteonecrosis and atypical femoral fractures [9]. Estrogen replacement therapy for postmenopausal women has been shown to be another effective osteoporosis therapy. Menopause, typified by reducing estrogen levels, is an important risk factor for osteoporosis. In 2020, the American Association of Clinical Endocrinologists (AACE) issued the “Guidelines for the Diagnosis and Treatment of Postmenopausal Osteoporosis,” in which intervention and treatment measures have been proposed on the basis of the etiology of postmenopausal osteoporosis [10]. Studies have shown that estrogen can affect bone remodeling by inhibiting osteoclast activity [11]. Although estrogen replacement therapy can effectively reduce menopause-associated osteoporosis risk, it is associated with life-threatening complications such as venous thrombosis and increased tumor development [12]. In light of the shortcomings of current therapies, it is necessary to continue studying the molecular mechanisms of osteoporosis in order to identify further new treatments. Different micro-RNAs (miRNAs) have been found to play important roles in the regulation of osteoblast and osteoclast activities [13]. Thus, miRNAs could be used as a potential biomarker and therapeutic target for osteoporosis. It is also possible to treat osteoporosis by harnessing the osteogenic differentiation ability of stem cells and their paracrine role in regulating cell function [14]. Thus far, stem cells have been used to treat osteoporosis in both rabbit and rat models [15], meaning the development of stem cell therapy in the clinical setting is imminent.

This article briefly summarizes the updates in the molecular basis of bone remodeling and the currently available treatment strategies for osteoporosis. More importantly, emerging new research directions are described, namely miRNAs, stem cells, bone marrow adipocytes, nerves and endothelium, gut microbiota, and bone targeting technologies, to shed light on future therapeutic avenues for this burdensome disease.

## The regulation of bone remodeling in health and osteoporosis

**Fig. 1:**
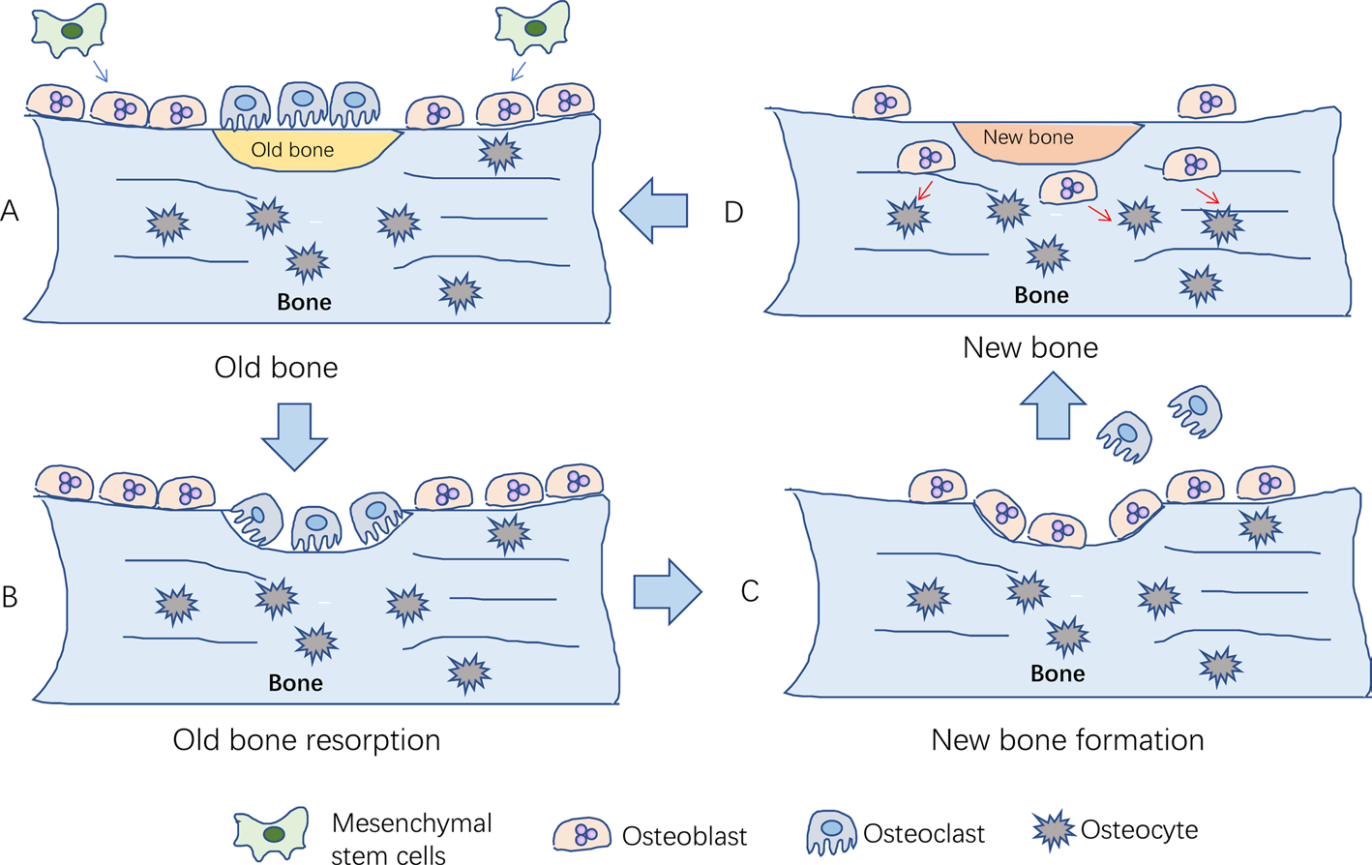
The process of bone remodeling under physiological conditions. **A** Local bone degenerates into old bone. Mesenchymal stem cells differentiate into osteoblasts; **B** osteoclasts migrate to the surface of old bone for bone resorption; **C** osteoclasts leave the surface after the old bone is absorbed, and then osteoblasts migrate to the surface for bone formation; **D** new bone replaces old bone to maintain bone quality, strength, and mass. After bone formation, osteoblasts differentiate into osteocytes

Osteoporosis results from an imbalance of normal bone remodeling, such that bone resorption is favored over bone formation. Human bones are stimulated by body weight, muscle traction, and high-intensity exercise. Over time, bones are damaged and degraded. Bone remodeling starts with bone resorption and ends with bone formation (Fig. 1). It is an essential process for maintaining mechanical strength, structural integrity, and mineralization by replacing old and damaged bone with new bone. However, the exact initial mechanisms underlying remodeling are yet to be fully elucidated. It is known that the process occurs in response to a number of factors, including hormone signals, paracrine and autocrine factors, and the physical pressure of mechanical loading [16]. Additionally, a range of systems —endocrine, immune, nervous, and more—are involved in the regulation of bone remodeling [17]. Environmental and genetic factors further influence this process; menopause, low BMI, white or Asian background, lack of sunshine, low exercise, malnutrition, disease, and certain drugs lead to bone microstructure damage and osteoporosis [18]. Although the exact mechanisms that initiate osteoporosis are yet to be fully elucidated, the signaling pathways that regulate bone resorption and formation have been extensively described. These are briefly outlined below. This is significant to note as most of the existing medications under development have focused on targeting such pathways, which mainly comprise mechanisms to control osteoclast and osteoblast action [19].

### Osteoclast differentiation and regulation of bone resorption

**Fig. 2:**
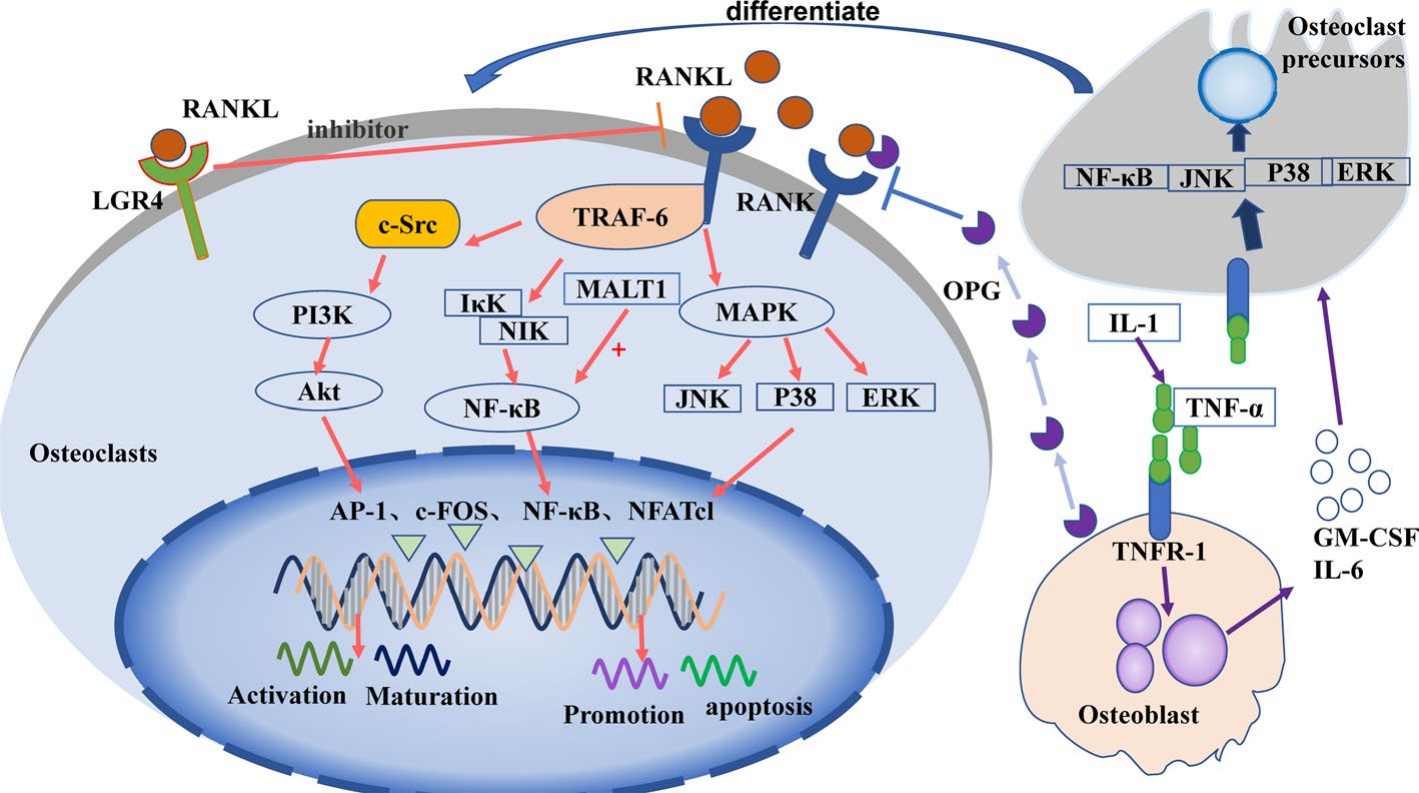
Signaling pathways in the control of osteoclast differentiation and maturation

Osteoclasts are the primary functional cells involved in bone resorption. They are granulocyte–macrophage colonies in the mononuclear macrophage system, formed by the fusion of monocyte precursors under the action of various factors secreted by bone marrow stromal cells [20]. Drawn by the action of chemokines, osteoclast precursors enter circulation and reach bone tissue in the absorptive state. These precursors are then induced to differentiate into osteoclasts by granulocyte macrophage colony-stimulating factor (GM-CSF) and RANKL. Mature osteoclasts then cover the surface of the absorbed bone tissue and release osteolysis-related enzymes for bone resorption [21]. It has been well documented that various factors affect bone resorption, including hormones, cytokines, and noncoding RNAs, by acting on signaling pathways in osteoclast differentiation. Among these signaling pathways, the RANKL/RANK/OPG and IL-1/TNF-α pathways are known to be critical for osteoclastogenesis, described below (Fig. 2).

#### RANKL/RANK/OPG signaling pathway

The RANKL/RANK/OPG signaling pathway is one of the most studied signaling pathways in bone homeostasis. It is essential to normal physiology, functioning to potently promote osteoclast differentiation and activity [22]. After being secreted by osteocytes, RANKL binds to the RANKL-specific receptor (RANK) on osteoclasts to upregulate their differentiation and activation [23]. Osteoprotegerin (OPG), a decoy receptor, is mainly produced by osteoblasts, and competes with RANKL to negatively regulate osteoclast differentiation [24].

RANKL binds to RANK to form a trimer, which then binds molecules to recruit tumor necrosis factor receptor-related factor-6 (TRAF-6). TRAF-6 passes through NF-κB inhibitor-κ-binding kinase (IκK) and NF-κB-induced kinase (NIK), causing them to activate NF-κB, which regulates osteoclast maturation, differentiation, or apoptosis [25]. TRAF-6 also activates c-Src [26], which stimulates phosphatidylinositol 3-kinase (PI3K). PI3K activates protein kinase B (PKB, Akt), which subsequently regulates osteoclast differentiation [27]. Additionally, RANKL/RANK activates the mitogen-activated protein kinase (MAPK) signaling pathway via extracellular regulated protein kinases (ERK1/2), c-Jun N-terminal kinase (JNK), or P38MAPK. The MAPK pathway results in the activation of transcription factors c-fos, activator protein-1 (AP-1), and nuclear factor of activated T cells-1 (NFATc1) [28], which then regulate the expression of matrix metalloproteinases (MMPs) [20] to stimulate the differentiation of osteoclast precursors into osteoclasts [29]. Recent studies suggest that protein phosphatase 2A (PP2A) promotes the expression of RANKL [30]. In addition, the leucine-rich G-protein-coupled receptor 4 (LGR4) was recently identified as another receptor of RANKL [31]. This is thought to competitively bind RANKL, thereby inhibiting the classical RANKL–RANK signal transduction pathway during osteoclast differentiation.

#### IL-1/TNF-α signaling pathway

IL-1 can induce tumor necrosis factor-α (TNF-α) to stimulate osteoblasts to produce granulocyte macrophage colony-stimulating factor (GM-CSF) and IL-6 [32], and induce osteoclast precursors to differentiate into osteoclasts [33]. TNF-α can also bind to TNF receptor-1 (TNFR-1) of osteoclast precursors, activate NF-κB, JNK, p38, or ERK, and promote the differentiation of osteoclast precursors into osteoclasts [34].

#### MALT1 signaling pathway

Mucosa-associated lymphoid tissue lymphoma translocation factor 1 (MALT1) regulates the NF-κB–NFATc1 signaling pathway and promotes osteoclast activation [35]. Following studies have shown that inhibitors of MALT1 inhibit NF-κB in osteoclasts, thereby strongly inhibiting the expression of NFATc1 and reducing osteoclast differentiation [36].

In osteoclasts, RANKL binds to RANK, then activates PI3K/Akt, NF-κB, or MAPK signaling via the recruitment protein TRAF-6, further activating transcription factors, such as AP-1, c-fos NF-κB, and NFATc1 to regulate osteoclast function. LGR4 inhibits the RANKL/RANK signaling pathway by binding to RANK. Osteoblasts secrete OPG to inhibit RANKL signaling and release GM-CSF or IL-6 to promote the differentiation of osteoclast precursors after induction with IL-1 or TNF-α.

### Signal pathways controlling osteoblast proliferation and differentiation

**Fig. 3:**
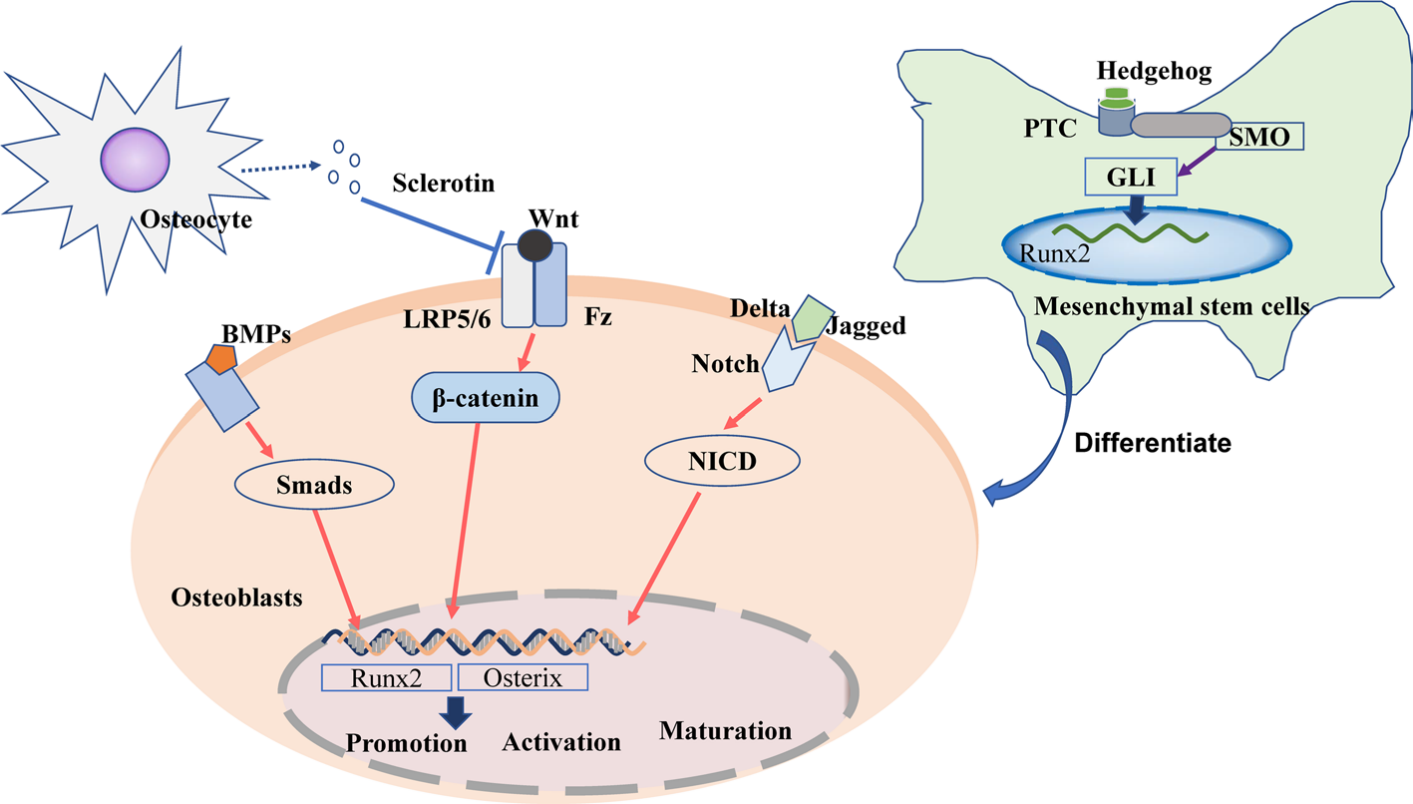
Signaling pathways regulating osteoblast differentiation and maturation

As with osteoclasts in bone resorption, osteoblasts are the major functional cells of bone formation. The precursor cells of osteoblasts are multipotent bone marrow mesenchymal stem cells (BM-MSCs), capable of several different cell lineages including osteoblasts, adipocytes, and chondrocytes [37]. After being stimulated to differentiate into osteoblasts, they are deposited on the bone surfaces. Here, they encourage bone formation and strength by synthesizing and secreting collagen, and promoting the mineralization of inorganic phosphorus and calcium ions to form hydroxyapatite. Osteoblasts may remain as bone-lining cells, or they can also be embedded in the bone matrix, at which point they become osteocytes. After repeating this process of osteoblast deposition and embedding multiple times, a new bone matrix is formed [38]. In terms of stem cell osteogenic differentiation and osteoblast activation, the most studied signaling pathways include the Wnt/β-catenin, BMP–Smad, Hedgehog, and Notch signaling pathways (Fig. 3).

#### Wnt/β-catenin signaling pathway

The Wnt signaling pathway includes both canonical and noncanonical pathways [39]. Of these two pathways, the canonical Wnt signaling pathway has been shown to play a particularly important role in osteoblastic bone remodeling [40]. The binding of Wnt protein in osteoblasts to low-density lipoprotein receptor-related proteins (LRP5/6) and Frizzled (Fz) receptors, located on the osteoblast membrane, promotes the stabilization of intracellular β-catenin [41]. β-Catenin can then translocate into the nucleus and regulate the expression of osterix and Runt-related transcription factor 2 (Runx2). These are key bone-specific transcription factors for osteogenesis [42], which thereby influence osteoblast activity [43].

#### BMP–smad signaling pathway

Bone morphogenetic proteins (BMPs) are important members of the transforming growth factor-β superfamily [44]. BMP2, 4, 7, and 9 play important roles in the differentiation of osteoblasts [44]. They bind to specific receptors on the cell membrane to phosphorylate downstream Smad proteins [45] (such as Smad1 and 5) and then further activate transcription factors [46], including Runx2 and osterix [47].

#### Hedgehog signaling pathway

The Hedgehog (Hh) signaling pathway is composed of Hh-corresponding ligands (IHH, Shh, DHH), receptors (Patched PTC, SMO), and intracellular signaling molecules (e.g., GLIs) [48]. After Hh binds to the PTC and SMO receptors on mesenchymal stem cell (MSC) membranes [49], it activates GLIs, which are translocated into the nucleus to upregulate the expression of the downstream target  Runx2 [50]. This results in MSC differentiation into osteoblasts instead of adipocytes [51].

#### Notch signaling pathway

The role of the Notch signaling pathway in skeletal metabolism is not always consistent. Jagged and delta-like proteins, such as Notch ligands, have been found to bind Notch and promote the translocation of the intracellular domain of Notch (NICD) into the nucleus, thus promoting osteoblast differentiation in vitro [52]. However, some studies have shown that NOTCH1 inhibits osteoclastogenesis, NOTCH2 enhances osteoclast differentiation [53], and NOTCH3 is the main signal of Notch signaling in osteoblasts [54]. As such, the role of the Notch signaling pathway in bone remodeling requires further elucidation.

In bone-forming osteoblasts, Wnt binds to the LRP5/6 or Fz receptors, induces β-catenin translocation into the nucleus, and activates the expression of osterix and Runx2 to regulate the promotion, activation, and maturation of osteoblasts. BMPs promote Smad phosphorylation to activate the expression of osterix and Runx2. Jagged and delta-like proteins bind to Notch, induce NICD translocation into the nucleus, and activate the expression of osterix and Runx2. Sclerotin secreted by osteocytes inhibits Wnt binding to osteoblasts. In bone marrow mesenchymal stem cells (BM-MSCs), Hh binds to the PTC and SMO receptors and activates GLIs that translocate into the nucleus to upregulate the expression of Runx2, promoting MSC differentiation into osteoblasts.

### Nutrients and known regulatory factors in modulating bone remodeling

The occurrence and development of osteoporosis is closely related to factors that regulate bone remodeling such as calcium, vitamin D, estrogen, and parathyroid hormone (PTH). As such, these factors have formed the basis for current pharmacological treatment strategies. The recent emergence of drugs targeting cytokines, such as RANKL and OPG, which regulate osteoclast activity, indicates how osteoporotic therapy has entered a new frontier steeped in molecular biology.

#### Calcium and vitamin D

Ninety-nine percent of the body’s calcium is stored in the bones. Sufficient calcium intake is essential for maintaining bone mass and strength. This is also dependent on sufficient intake and activation of vitamin D, which promotes effective absorption of intestinal calcium. Active vitamin D [1,25 (OH)2D3] also directly promotes bone health by binding to vitamin D receptors (VDRs) in bone cells to regulate bone remodeling [55]. To be converted into its active form [56], it is hydroxylated twice, first in the liver and then in the kidneys. 1,25(OH)2D3 can promote both osteoclast activity, by influencing RANKL and NFATc1 signaling [57], and osteogenic activity, through BMP-2, Smad, Runx2, and the Wnt pathway [58].

In addition to its essential role in bone health, calcium, in the form of ionized calcium, is critical in a number of physiological functions, including neuronal function, muscle contraction, clotting, and intracellular signaling. Organisms cannot survive when such functions are compromised. As such, in conditions of low circulating calcium, bone undergoes increased resorption in order to supply circulating calcium ions for these life-sustaining functions. This is achieved predominantly via PTH [59], which stimulates bone resorption and increases the renal formation of active vitamin D to increase calcium absorption [60]. Conversely, calcitonin (CT) is a negative regulatory hormone of calcium. Secreted by thyroid C cells, CT inhibits the absorption of calcium from the intestine, promotes the excretion of calcium from the kidneys, and inhibits bone resorption, thereby reducing blood calcium levels [61].

#### Estrogen

Estrogen is critical for maintaining bone homeostasis. Its action is mediated primarily by the estrogen receptors ERα and ERβ, which are expressed in a variety of cells. Such receptors have been found to be widely expressed in osteocytes, osteoblasts, BM-MSCs, and osteoclasts. However, it is generally believed that estrogen’s bone-related activity occurs predominantly by influencing bone resorption to regulate bone remodeling [62]. Estrogen inhibits the secretion of RANKL and promotes the secretion of osteoclast-inhibiting factors such as growth hormone, GLP-1, and osteoprotegerin (OPG), thereby inhibiting osteoclast activity [11]. In addition to its primary role in inhibiting bone resorption, estrogen promotes osteogenic differentiation of MSCs and maintains the number of osteoblasts [11]. Therefore, estrogen deficiency, as in postmenopausal women, can lead to bone loss, which eventually progresses to osteoporosis. Hormone replacement therapy (HRT) has proven effective at preventing bone loss in postmenopausal women. In men [63], studies have found that testosterone can regulate bone metabolism directly and by being converted to estrogen [64]. Indeed, inhibition of aromatase, the enzyme responsible for androgen conversion into estrogen, resulted in decreased BMD in male rats [65].

#### Cytokines regulating bone metabolism

Cytokines provide another mechanism by which regulatory factors such as PTH and estrogen modulate bone remodeling, whereby such factors induce cells to release cytokines. A number of cytokines are involved in the regulation of bone metabolism, produced by bone cells themselves, as well as inflammatory cells and more. Osteoblasts secrete RANKL and OPG, as well as IL-1, IL-6, and TGF-β, to regulate the differentiation, activity, and apoptosis of osteoclasts [33]. Sclerotin, secreted by osteocytes, can prevent Wnt from binding to LRP5/LRP6, resulting in a decrease in β-catenin, thereby inhibiting bone formation [66]. Elsewhere, macrophages, endothelial cells, and fibroblasts secrete RANKL [67] and macrophage colony-stimulating factor (M-CSF) [68]. Knowledge of the cytokines that regulate bone metabolism has led to the development of novel osteoporosis treatments currently used in clinical settings, including RANKL monoclonal antibodies, sclerotin monoclonal antibodies, and cathepsin K inhibitors.

### Emerging regulatory factors for bone remodeling

Our understanding of the factors that regulate bone remodeling is growing on a molecular, cellular, and whole organism level. In addition to the factors described above, increasing evidence has shown that noncoding RNAs [69], stem cells [14], bone marrow adipocytes [70], neuromodulation [71], exosomes [72], and gut microbiota [73] can also affect bone remodeling and participate in the process of bone metabolism. These factors may also underpin novel therapeutic avenues for osteoporosis, but their potential for translation into clinical applications is yet to be tested.

#### MicroRNAs

MicroRNAs (miRNAs) are noncoding, single-stranded RNA molecules encoded by endogenous genes to play a role in regulating posttranscriptional gene expression within bone cells. Namely, they regulate the expression of functional proteins in the bone activity signaling pathway [69]. Studies have found that other noncoding RNAs, such as long-noncoding RNAs (lncRNAs) and circular RNAs (circRNAs), are involved in the regulation of bone metabolism; however, miRNAs are the main subject of extensive and in-depth research [74]. For example, miRNA-21 (miR-21), which can be upregulated by RANKL, activates the PI3K/Akt signaling pathway by targeting PTEN [75] (a homologous gene of phosphate and tension on chromosome 10). This results in the promotion of osteoclastogenesis and bone resorption [76].

The relative activity and profile of miRNAs can differentially affect skeletal health and osteoporosis. Studies have shown that miR-31 [77], miR-103-3p [78], and miR-29b-3p [79] downregulate osteoblastic activity by inhibiting the expression of Runx2 [80]. Negative regulation of osteoblasts is also performed by miR-9-5p [81], miR-124 [82], and miR-203a-3p [83], which inhibit signal transduction of the Wnt signaling pathway, and miR-100 [84], which inhibits BMP signaling pathways. On the other hand, miR-194 [85], miR-874 [86], miR-96 [87], and miR-135-5p [88] can promote osteoblastic activity by stimulating Runx2, Wnt, and other molecules. Osteoclast activity is promoting by miR-21 [89], miR-183 [90], miR-155 [91], mir-148a [92], and miR-214 [93], which can inhibit the expression of RANKL, PI3K, TNF-α, and other molecules. miR-17 [94], miR-29 [95], and miR-503 [96] can downregulate osteoclast activity by inhibiting the RANKL signaling pathway. Elsewhere, miR-200a-3p [97], miR-449b-5p [98], and miR-579-3p [99] inhibit osteogenic differentiation of MSCs by affecting Runx2, chemokine receptor (CXCR), and other signaling pathway molecules. Studies investigating the wide-ranging effects of different miRNAs (Table 1), involving animal models and human cohort studies, have outlined their promise as a therapeutic target for osteoporosis. However, the translation to a clinical application is yet to be tested.

**Table 1:** The effects of microRNAs (miRNAs) on the activity of bone cells

MicroRNA	Targets	Cell activity	Experimental models	References
miR-31, miR-103-3p, miR-133, miR135a-5p, miR-203a, miR-375, miR-29b-3p	Runx2	Inhibit osteoblast activity	Serum, cell	[77–79, 83, 100, 101]
miR-9-5p, miR-124, miR-203a-3p	Wnt/β-catenin		Serum, cell	[81–83]
miR-100	BMP/Smads		Serum, cell	[84]
miR-542-3p, miR-543	PI3K/AKT		Serum, cell, rats	[102, 103]
miR-194, miR-874	Runx2	Promote osteoblast activity	Serum, cell, rats	[85, 86]
miR-96	Wnt/β-catenin		Serum, cell, mice	[87]
miR-216a	BMP/Smads		Serum, cell	[104]
miR-216a	PI3K/AKT		Serum, cell	[104]
miR-21, miR-183, miR-155	RANKL	Promote osteoclast activity	Serum, cell, mice	[89–91, 105]
miR-21, miR-148a, miR-214	PI3K/AKT		Serum, cell, mice	[92, 93]
miR-155	TNF-a, IL-1		Serum, cell	[91]
miR-17, miR-29, miR-503	RANKL	Inhibit osteoclast activity	Serum, cell, mice	[94–96]
miR-124	NFATc1		Serum, cell	[105]
miR-200a-3p, miR-449b-5p, miR-579-3p	Runx2, CXCR, SLC, SIRT1	Inhibit MSC osteogenic differentiation	Serum, cell	[97–99]
miR-15b, miR-29b	Smads, PI3K/AKT	Promote MSC osteogenic differentiation	Serum, cell	[98]

While the regulation of bone remodeling by miRNAs is a hot topic worthy of further clinical exploration, greater scientific knowledge is needed before entering the clinical application stage. Current miRNA research on osteoporosis is mostly limited to interventions at the cellular and animal levels. What is lacking is an in-depth exploration of the molecular interactions.

One such area of particular clinical interest for further study is the modulating effect of lncRNA and circRNA on miRNA. Both lncRNA and circRNA have miRNA-binding sites, which act as miRNA sponges in cells to counteract the inhibitory effect of miRNA on their target genes. Accordingly, this increases the expression level of target genes. This interaction can form a complex CeRNA (competing endogenous RNA) network, which plays an important role in various biological processes and disease progression. In osteoporosis, some lncRNAs and all circRNAs affect the differentiation of osteoblasts and osteoclasts by acting as miRNA sponges. The study of this interaction will help to analyze the pathogenesis of osteoporosis and the development of new drugs for the treatment of osteoporosis. For example, lncRNA TUR1 can further regulate osteoblast function by targeting PTEN as a synergistic effect of miR-21 [76]. circRNA-28313 can alleviate miR-195a by forming a CeRNA network to inhibit CSF1 (colony stimulating factor 1), functioning to regulate the osteoclast differentiation [13].

#### Stem cells

In addition to directly differentiating into osteoblasts, BM-MSCs can also act on osteoblasts and osteoclasts via a paracrine effect. Direct injection of stem cells in the treatment of osteoporosis is mainly found to operate via such paracrine mechanisms [106]. Stem cell therapy has been proven to be effective in animal model research. However, many problems remain to be solved to translate this treatment into clinical medicine, including stem cell extraction method, clinical ethics, allogeneic rejection, and so on. Stem cells have also been found to secrete exosomes as a means of intercellular regulation [72]. Exosomes are highly heterogeneous and contain a variety of proteins and RNAs. In light of their wide-ranging components and effects, their potential as an osteoporosis treatment requires further investigation.

#### Bone marrow adipocytes, bone endothelium, and bone nerves

In addition to bone itself, studies have also found that other tissues in bone, namely adipocytes, blood vessels, and nerves, can regulate bone remodeling. Bone marrow adipocytes may affect the development and function of other cell types in bone by secreting adipokines [70]. Some studies have reported that adipocyte conditioned medium samples inhibit the formation of osteogenic lineages of BM-MSCs and promote the formation of osteoclasts. Several key inhibitors of osteoblast differentiation have been identified as adipokines secreted by bone marrow adipocytes. Preadipocytes secrete curl-associated protein 1 (SFRP-1), which inhibits Wnt/β-catenin signals to reduce osteogenesis. Therefore, investigating ways to reduce the activity of bone marrow adipocytes and increase the proportion of bone marrow stem cells may hold promise as a new osteoporosis treatment [107]. Elsewhere, there is evidence that the growth of blood vessels in bone is coupled with osteogenesis [108]. Studies have found that bone endothelial cells secrete HIF-1α, which can affect bone angiogenesis and osteogenesis. Additionally, bone cells and bone endothelium and been observed to have a complementary interaction whereby osteoblasts release proangiogenic factors, which promotes angiogenesis and subsequently improves skeletal health [109]. SLIT3 was determined to be an osteoblast-derived angiogenic factor through transcriptome analysis [110]. In a postmenopausal osteoporosis mouse model, the use of recombinant SLIT3 can not only enhance fracture healing, but also offset bone loss. Other studies have found that nerve conduction signals in bone, such as cholinergic signals, may also be related to osteoporosis [111]. In osteoporotic rats, osteoblasts contained significantly decreased levels of muscarinic acetylcholine receptor (mACHR) M5 and M3. These findings provide evidence for the involvement of AChR signaling in osteoporosis [71]. This displays how intraosseous adipocytes, blood vessels, and nerves can all regulate bone metabolism and thus are implicated in the pathophysiology of osteoporosis. Although therapies based on this knowledge are not in the clinical stage, they may become a new treatment direction for the research and development of osteoporosis treatments.

#### Gut microbiota

Gut microbiota regulates human nutrition, metabolism, vitamin production, and immune system function, thus affecting bone metabolism [112]. Steroid hormones, PTH, and vitamin D metabolites may be affected by microbiota [73]. Additionally, compounds of bacterial origin, such as vitamins, may reach the blood and directly affect osteocyte activity. Further, the gut microbiota may affect host microRNAs (miRNAs) [113], such as miRNA-33-5p and miRNA-194, thereby influencing the development of osteoporosis [114]. Although this correlation between gut microbiota and bone metabolism has been found, whether bone physiology can be targeted through microbiota intervention requires further exploration. There are still many new targets being explored, such as platelet-derived growth factor-BB (PDGF-BB) [115] secreted by pre-osteoclasts, sphingosine-1-phosphate lyase [116], and integrin-β3 signaling [117], as some new research strategies are needed to enter clinical research.

### Current research strategies

Current research strategies to find new target factors for osteoporosis treatment involve investigating the genomics, proteomics, epigenetics, and metabolomics of human samples [69]. To date, a large number of osteoporosis-associated genome-wide association studies (GWAS) have been carried out to identify the genetic risks of osteoporosis [118]. Several single-nucleotide polymorphisms (SNPs) have been revealed from the GWAS to be associated with low BMD and increased risks of osteoporotic fracture [119]. Mechanistically, these SNPs are believed to increase osteoporosis susceptibility via influencing the binding affinity of transcriptional factors or miRNAs [13]. For example, the genetic association between RANKL and BMD was reported through human GWAS [118]. This link was then explored at a cellular and whole organism level using animal models, and lastly assessed for clinical application. As a result, the link between RANKL and BMD gave rise to the RANKL-targeting drug denosumab, currently used in clinical settings for postmenopausal osteoporosis. This series of studies on the RNAKL–OPG system also highlights the importance of utilization of animal models in osteoporosis research that leads to the identification of new therapies. Several types of animals, including mice, rats, dogs, rabbits, and nonhuman primates, have been utilized in osteoporosis research [120]. Ovariectomized models (simulating postmenopausal osteoporosis), aging [121] and glucocorticoid-induced models (mimicking human glucocorticoid osteopenia) [122], and retinoic acid (RA)-induced models [120] are among the most widely used animal models. The knowledge gained from these animal models provides critical in vivo physiological and pathological evidence that reflects bone function and health in humans [123]. Importantly, the knowledge of the etiology, prevention, and treatment of osteoporosis obtained from these animal studies [124] could lead to the identification of new regulatory factors that could be developed as early diagnostic biomarkers and therapeutic targets for osteoporosis [125].

## Pharmacologic strategies for osteoporosis

An understanding of the factors that regulate bone resorption and formation has allowed researchers to develop pharmacological agents to combat osteoporosis. Although there is a wide array of treatments available that have produced beneficial effects, many of these also come with disadvantages, as listed in Table 2. This necessitates further research that both evaluates the value of current treatments and explores new therapeutic avenues that hopefully yield higher efficacy with fewer adverse effects.

**Table 2:** Summary of drugs for osteoporosis treatment and their side effects

Category	Drug	Clinical drug name	Side effects
Bone basic nutrient supplements	Calcium	Calcium carbonate, calcium acetate	Hypercalcemia caused by overdose
	Vitamin D	Vitamin D, 1αOH-VitD, 1,25OH-VitD	Hypercalcemia and vitamin D poisoning caused by overdose
Antiresorptive	Bisphosphonate	Alendronate, zoledronic acid, sodium risedronate, ibandronate, etidronate, chlorophosphonate	Gastrointestinal adverse reactions, transient influenza-like symptoms, nephrotoxicity, mandibular necrosis, atypical femoral fracture
	Menopausal hormone	Estrogen, progesterone	Risk of estrogen-related diseases such as endometrial cancer, breast cancer, cardiovascular diseases, venous thrombosis, obesity
	Selective estrogen receptor modulators, SERMs	Raloxifene	Not suitable for male patients with osteoporosis. The risk of venous thrombosis is lower than that with estrogen
	Calcitonin	Elcatonin, salcatonin	Some cases of facial flushing, nausea, and allergy
	Fully human RANKL monoclonal antibody	Denosumab	Hypocalcemia, infection (cystitis, upper respiratory tract infection, pneumonia, skin cellulitis, etc.), rash, skin pruritus, muscle or bone pain; long-term application may over-inhibit bone resorption, resulting in mandibular osteonecrosis or atypical femoral fracture
	Cathepsin K inhibitor	Odanacatib	Cardiovascular events including atrial fibrillation and stroke risk
Anabolic	PTH analogues	Teriparatide acetate, abaloparatide	Short-term hypercalcemia, the treatment time should not exceed 24 months,
	Anti-sclerotin monoclonal antibody	Romosozumab	Need further clinical data
	Vitamin K	Menatetrenone	Stomach discomfort, contraindicated for patients taking warfarin
Bidirectional regulation	Strontium	Strontium ranelate	Venous thrombosis risk, adverse reactions of cardiovascular and cerebrovascular diseases

### Bone nutritional supplements: calcium and vitamin D

Adequate calcium intake is protective against osteoporosis and associated osteoporotic fractures. Calcium supplementation prevents the mobilization of bone calcium into the blood, reducing bone resorption and thus slowing bone loss [126]. Bone formation requires sufficient calcium to obtain an ideal bone peak, improve bone mineralization, and maintain bone health. Therefore, calcium supplements are a simple first-line treatment for osteoporosis. There is minimal risk of adverse effects, especially as the dosage should be adjusted according to the calcium intake of the population so as to prevent hypercalcemia [127]. However, calcium supplements alone cannot be used for the treatment of osteoporosis [128].

Vitamin D facilitates calcium absorption and can act directly on osteoblasts and osteoclasts to promote bone mineralization and inhibit bone resorption [129]. Studies have shown that vitamin D can prevent sarcopenia, improve muscle strength and postural stability, and reduce the risk of falls. Therefore, as with calcium, vitamin D is an essential nutrient for the treatment of osteoporosis, whose supplementary dose should be adjusted according to the vitamin D levels of the target population. Elderly individuals over 60 years of age need to increase their intake of vitamin D owing to a lack of sunshine and malabsorption of vitamin D. At present, vitamin D drugs include vitamin D, 25-hydroxy-vitamin D and 1,25 hydroxy-vitamin D. Hydroxy-vitamin D does not need to be activated by the liver and kidneys and can directly act on target organs. The effect of hydroxy-vitamin D is better than that of pure vitamin D, and it can also be taken by those with coexisting liver and/or kidney disease [130]. However, with this added benefit comes a higher financial cost. Similar to calcium supplements, the effect of vitamin D on osteoblasts and osteoclasts is not sufficient to treat osteoporosis on its own. Vitamin D needs to be used in combination with calcium and other anti-osteoporosis drugs. It is worth noting that excessive vitamin D intake can increase the blood calcium concentration above physiological levels. As a result, blood calcium can precipitate out as deposits in other organs and tissues, such as renal calcification, or even in the brain, causing deleterious effects [131].

### Medications to inhibit bone resorption

Antiresorptive agents are currently the mainstay of osteoporosis treatment. They inhibit osteoclast activity by targeting a variety of processes involved in osteoclast function, thereby reducing bone resorption. These include bisphosphonates, estrogen, calcitonin, cathepsin K inhibitors, and RANKL inhibitors (Table 2).

#### Bisphosphonates

Bisphosphonates (BPs) are the first-line treatment for osteoporosis [132], taken in combination with calcium supplements [10]. BPs combine with hydroxyapatite on the bone surface, preventing cytokine release that would normally activate osteoclasts. As a result, osteoclasts cannot interact with the bone and they undergo increased apoptosis, causing reduced bone resorption [6]. This corresponds to effective clinical outcomes such as improved BMD and reduced rates of osteoporotic fractures [132]. Bisphosphonate drugs include both oral and intravenous forms. Given their proven results, many bisphosphonates are currently used in clinical settings: alendronate, zoledronate, risedronate, ibandronate, etidronate, and chlorophosphonate. However, studies have shown that long-term use of BPs may inhibit bone turnover and increase bone brittleness [133]. In addition, for patients with long-term use of BPs (usually > 3 years, with a median treatment time of 7 years), excessive inhibition of bone resorption can increase the risk of mandibular osteonecrosis or atypical femoral fracture [134].

#### Estrogen-related therapy

Estrogen replacement therapy (ET) and estrogen plus progesterone therapy (EPT) have been demonstrated to reduce bone loss and the risk of osteoporotic vertebral, nonvertebral, and medullary fractures in postmenopausal women [12]. Common estrogenic drugs are divided into natural and synthetic drugs. Natural estrogen drugs include estradiol, estriol, and estrone. Synthetic estrogen drugs include ethinylestradiol, ethinylether, and estradiol valerate, which have long-lasting effects. While estrogen replacement therapy is effective in reducing the risk of osteoporosis during menopause, long-term use of estrogen has been associated with increased risk of serious diseases [135] such as endometrial cancer, breast cancer, venous thrombosis, and stroke [136]. Combining this with progesterone, as in EPT, can alleviate some of these risks, particularly for endometrial cancers.

Selective estrogen receptor modulators (SERMs) provide another way of delivering the beneficial effects of estrogen replacement therapy while reducing estrogen-associated risks. SERMs bind to estrogen receptors in different tissues and, depending on the tissue type, can either produce agonistic or antagonistic biological effects [137]. For example, the SERM raloxifene has been found to play an agonistic role in bone tissue, where it inhibits bone resorption, increases bone density, and reduces the occurrence of vertebral fractures in postmenopausal women [138]. On the other hand, it has antagonistic effects on breast and uterine estrogen receptors [139]; by not stimulating breast or uterine tissue, it reduces the incidence of estrogen receptor-positive breast cancer and endometrial cancer [140]. This highlights a significant advantage of SERMs over traditional estrogen therapy. The use of SERMs in men has also been met with interest; however, it has so far been fraught with side effects and requires further exploration before clinical application [140].

#### Calcitonin for treatment

Calcitonin drugs used to treat osteoporosis include salcatonin and carbocalcitonin, which are extracted from salmon and eels. In addition to regulating calcium metabolism, calcitonin can also inhibit osteoclast proliferation and directly bind to them via calcitonin receptors to reduce osteoclast activity [61]. Administration of exogenous calcitonin inhibits bone resorption and improves BMD in patients with osteoporosis [141]. Furthermore, within the effective dose, combined with calcium and vitamin D supplementation, exogenous calcitonin does not reduce blood calcium levels. Within osteoporosis treatment, calcitonin has often been used more specifically to alleviate bone pain induced by osteoporosis. This benefit is activating endogenous opioid system and increasing β-endorphin concentration in the blood, providing analgesic effects. It can also inhibit the production of prostaglandins in local inflammatory tissues that act directly on the central nervous system pain receptors to produce analgesic effects [142].

#### Cathepsin K inhibitors

Cathepsin is a protease found in the cells (especially within lysosomes) of various animal tissues that hydrolyze proteins. Cathepsin K is a member of the cathepsin family and is expressed by osteoclasts, mainly functioning to degrade type I collagen in bone tissues [143]. It also promotes the inactivation and degradation of non-collagen factors, such as osteocalcin, osteopontin, osteonectin, proteoglycan, and related growth factors in bone tissue. The cathepsin K inhibitor odanacatib (ODN), developed by Merck (USA), inhibits this degradation of the bone matrix to treat osteoporosis [144]. Recent studies have found that ODN can increase the cortical thickness and bone mineral content of trabecular bone, thereby increasing BMD and bone load strength [145]. However, according to the long-term odanacatib fracture trial (LOFT), conducted at 388 centers across 40 countries involving over 16,000 participants [146], ODN was associated with significantly higher rates of atrial fibrillation and stroke. Owing to its unfavorable benefit–risk profile, it is rarely used clinically.

#### RANKL inhibitors

RANKL is one of the most important molecules involved in the regulation osteoclast activity. Denosumab, developed by Amgen (USA), is a fully human RANKL monoclonal antibody that prevents RANKL from activating its receptor on osteoclasts and pre-osteoclasts, leading to the inhibition of bone resorption and a subsequent increase in bone mass. Compared with BPs, denosumab can improve BMD more quickly, including in cortical and cancellous bone, and reduce the risk of fracture [7]. Clinical studies have found that an increase in bone density can still be observed after 10 years of denosumab treatment, which is better than that observed with BP drugs. However, studies have shown that denosumab discontinuation causes a rapid decline in BMD due to a rebound activity in osteoclasts, leading to an increase in the incidence of multiple vertebral fractures [8]. This phenomenon is called “drug holiday” [147]. It is suggested that denosumab should be used continuously if it is tolerated, and in the event of discontinuation, a stepwise approach or combination with other therapies such as bone-forming drugs should be considered to reduce or prevent rebound bone loss and fracture [147]. Similar to using bisphosphonates, long-term use with denosumab will still increase the risk of mandibular osteonecrosis and atypical femoral fractures, owing to excessive inhibition of bone resorption [148].

### Drugs that promote bone formation

Compared with antiresorptive drugs, there are fewer osteoporosis medications on the market that work by promoting bone formation. However, such drugs that target osteoblasts and operate via anabolic actions are described below and summarized in Table 2.

#### Parathyroid hormone (PTH) analogues

PTH promotes bone resorption when blood calcium levels decrease [59]. However, intermittent low-dose use of PTH analogues (PTHA) has been shown to stimulate osteoblast activity and promote osteogenic activity [149]. As the dose increases, it can also stimulate osteoclast activity, inducing bone resorption instead [150]. Teriparatide is an active fragment of recombinant human PTH 1–34 (rhPTH1–34) [151]. Treating osteoporosis with teriparatide alone causes the bone metabolic rate to increase significantly in the first 6 months. This corresponds with an increase in bone mass, especially cortical bone resorption holes, but also a transient decrease in bone strength, especially in the hip bone [151]. As such, PTHAs are suitable for patients with vertebral fractures or extremely low bone density, where PTHAs can quickly increase bone density, but they must be combined with BPs to maintain bone density long term [152].

#### Anti-sclerotin antibody

Sclerotin is secreted by osteocytes and inhibits bone formation by inhibiting the Wnt signaling pathway y[153]. Romosozumab is a monoclonal antibody against sclerotin, which was developed by Amgen (USA) and approved by the US Food and Drug Administration (FDA) in 2019 [66]. It improves osteoporosis by reducing sclerotin expression or inhibiting its effect on the Wnt signaling pathway in osteoblasts [66]. In some countries, including Japan and Germany, it has now entered clinical applications for the treatment of osteoporosis in postmenopausal women with a high fracture risk [154]. In a phase III trial, compared with placebo and oral alendronate, the use of romosozumab for 12 months significantly reduced the risk of vertebral body and clinical fractures in postmenopausal women with osteoporosis. After further follow-up for 12–24 months, the risk of fracture also improved significantly [155]. However, owing to the short clinical application time, there are insufficient clinical data to fully evaluate the efficacy and side effects of this drug.

### Drug targets with bidirectional regulation

Strontium is a trace element in the human body, almost entirely located in bone [156]. Strontium exerts an anti-osteoporotic effect by promoting osteoblasts, inhibiting osteoclasts, and regulating MSCs[157]. Strontium ranelate (SrR) is a strontium salt drug used clinically [158], proven to be more effective in treating postmenopausal osteoporosis than 25-hydroxy-vitamin D [156]. However, SrR can cause a number of adverse reactions, including skin damage, ischemic heart disease, peripheral vascular disease, and cerebrovascular disease [159]. This is a major reason why SrR is not widely used in the treatment of osteoporosis [160].

### Potential novel therapeutic targets for osteoporosis

Despite having a variety of drugs available on the market, current pharmacological treatments for osteoporosis are either relatively ineffective or unsafe. Therefore, new treatments that produce better clinical outcomes with fewer adverse effects are urgently needed. In recent years, treatments based on stem cells and miRNA, as well as bone-targeting methods, have received increasing interest as novel therapeutic avenues for osteoporosis.

#### Stem cell therapy

Stem cell therapy is an emerging new treatment approach that harnesses stem cells’ great potential to differentiate and regulate intercellular communication. The stem cells used for research can come from different sources, including embryonic stem cells (ESCs), adult stem cells (ASCs), and induced pluripotent stem cells (iPSCs) [161]. So far, stem cell therapy has provided a great opportunity for degenerative disease and diseases that require tissue regeneration, such as stroke, premature ovarian failure, and spinal cord injury [162]. The main aim of stem cell therapy in osteoporosis treatment is to promote bone formation, rather than reducing resorption [163]. This is currently being explored via many in vivo animal studies as summarized in Table 3. Stem cells have the ability to differentiate into osteoblasts, promote the growth of osteoblasts, and inhibit the activity of osteoclasts through cell-to-cell interactions, using cytokines, chemokines, and extracellular vesicles [14]. From these actions, stem cells can reverse degenerative damage to bone by improving cell lifespan and activity. Currently, this therapy is still in the early stages of cell and animal experimentation. In rat and rabbit models, injection of stem cells can improve the microstructure of osteoporotic bone tissue, increase bone density, and increase the osteogenic activity of alkaline phosphatase (ALP) and osteocalcin (OCN) [164]. Additionally, the injection of stem cells can also promote the expression of OPG and inhibit TNF-α and RANKL, demonstrating improved osteogenic differentiation ability [165]. Owing to ethical issues, it is difficult to conduct experiments with ESCs and iPSCs in human research. Adult stem cells such as BM-MSCs, adipose stem cells (ADSCs), and hematopoietic stem cells do not involve such ethical barriers, and are also highly available [166] and amenable to clinical transformation [167]. However, their differentiation potential is generally weaker than that of ESCs, and the problem of stem cell homing after injection remains unsolved. At present, in animal models, the chemotactic ability of implanted stem cells can be improved by overexpression of chemokines such as CXCR4 or RANK-Fc [168]. Another shortcoming of stem cell treatment is the unwanted differentiation of transplanted MSCs and their potential to suppress antitumor immune responses, in addition to generating new blood vessels that may promote tumor growth and metastasis [162]. According to previous reports [169], stem cells have been used to treat human patients with osteogenesis imperfecta. To further develop stem cell therapy as a bona fide clinical treatment for osteoporosis, it will be necessary to increase its safety, especially in relation to their oncogenic effects [162].

**Table 3:** In vivo animal experiments involving different types of stem cell for osteoporosis treatment

Classification	Cell type	Cell modification	Animal model	Route of administration	Indicators	Reference
Embryonic stem cells	ESCs	Collagen I matrix implant	Femur fracture in OVX mice	Injection to bone surface	BMD, microCT	[170]
Adult stem cells	Bone marrow MSCs	Cells with GFP	OVX mice	Intravenous injection	BMD, microCT	[166]
		Cells with RANK-Fc or CXCR4 overexpression	OVX mice	Intravenous injection	BMD, microCT	[168]
		PLGA/CoI microspheres combined	OVX rat	Intra-bone marrow injection	BMD, microCT	[171]
	Human VSELs	Collagen sponge scaffolds	Cranial defects generated in SCID mice	Injection to bone surface	BMD, microCT	[172]
	UCB-MSCs	Nanofiber-expanded CD34^+^ cells	Glucocorticoid-induced NOD/SCID mice	Intracardiac ventricular injection	BMD, microCT	[173]
	ADSCs	Zfp467 siRNA transfection	OVX mice	Intravenous injection	BMD, microCT	[174]
		Collagen I matrix implant	OVX rabbit	Intra-bone marrow injection	BMD, microCT	[167]
		Young and aged ADSCs	Ovariectomized SAMP8 female mice (4 months of age)	Intra-bone marrow injection	BMD, microCT	[165]
Induced pluripotent stem cells	iPSCs	Calcium phosphate cement (CPC) scaffold	Cranial bone defect model in nude rats	Injection to bone surface	BMD, microCT	[175]

*ESCs* embryonic stem cells, *MSCs* mesenchymal stem cells, *VSELs* very small embryonic-like cells, *UCB-MSCs* umbilical cord blood MSCs, *ADSCs* adipose-derived mesenchymal stem cells, *iPSCs* induced pluripotent stem cells, *OVX* ovariectomized, *NOD/SCID* nonobese diabetic/severe combined immunodeficient, *GFP* green fluorescent protein, *RANK-Fc* receptor activator of nuclear factor-κB-Fc, *CXCR4* CXC chemokine receptor-4, *PLGA/CoI* polylactic acid polyglycolic acid copolymer (PLGA)/collagen type I (CoI), *Zfp467* zinc finger protein 467 *BMD* bone mineral density

#### miRNA-based therapy

miRNA-based therapy has shown potential in the treatment of osteoporosis and osteoporotic fracture [176]. There are abundant in vitro studies in which miRNA mimetics or inhibitors have been used to treat MSCs, osteoblasts, or osteoclasts [177] to determine the relationships between miRNAs and bone cell activity [178]. However, few in vivo studies have been conducted. Current in vivo experiments have mostly used miRNA inhibitors, lentiviral transfection, or exosomes to intervene in rat or mouse osteoporosis models. Following this, the bone quality of the rodents was observed to determine whether the regulation of miRNA influenced osteoporotic progression [87]. The clinical translation of lentivirus transfection can be difficult, but the clinical transformation of inhibitors is possible. Currently, the most widely used miRNA inhibitors are modified nucleoside oligomers, such as anti-miRNA oligonucleotides (AMOs) [179], locked nuclear acid (LNA) AMOs, antagomirs [180], and miRNA sponges [181]. The binding of miRNAs and target mRNAs is competitively inhibited using the principle of complementarity with the target miRNA sequence. However, the exploration of chemical small-molecule inhibitors for miRNA is still in its infancy, and few are used in osteoporosis models. For example, studies have shown that, compared with the ovariectomized (OVX) group, the BMD of rats treated with miR-30a-3p inhibitors was significantly increased; the miR-30a-3p inhibitor significantly upregulated bone volume/total volume (BV/TV), trabecular number (TB. N), and trabecular thickness (TB. Th) in OVX rats [182]. Since miRNAs can affect multiple signaling pathways, this could result in off-target activity and corresponding adverse effects. Therefore, further research is needed to develop tissue-specific miRNA inhibitors for osteoporosis treatment.

#### Bone-specific targeting technology

Regardless of the drug class, the ability to target bone specifically remains a highly significant barrier to overcome in order to advance osteoporosis therapy [183]. Owing to their ability to specifically bind with hydroxyapatite, bisphosphonates have been engineered for combination drug use, whereby they act like a vehicle to help other agents target bone tissue. Previous studies have combined iron oxide nanoparticles with bisphosphonates to deliver them to bone tissue. The iron oxide nanoparticles then exert their anti-osteoporotic action by removing active oxygen in bone to promote osteogenesis, which also occurs synergistically with bisphosphonates’ antiresorptive effect [184]. Elsewhere, exosomes have shown promise in providing bone-specific targeting. Exosomes secreted by BM-MSC have been engineered to both contain siSHN3 and modify the bone-targeting peptide. This enables them to specifically combine with osteoblasts to promote the expression of SLIT3 (vascular endothelial growth factor). As mentioned earlier, SLIT3 can not only enhance fracture healing, but also offset bone loss to treat osteoporosis [185]. In stem cell therapy, the overexpression of chemokine CXCR4 in transplanted MSCs has also been shown to improve the stem cell tracking to bone [168]. This displays how engineering bone-targeting technologies is an important branch of osteoporosis research, with the potential to produce highly specific and effective treatments.

## Conclusions and outlook

Bone remodeling requires a finely tuned balance of bone resorption and formation to maintain bone health. The control of this process involves an orchestrated web of regulation at a molecular and cellular level. When the bone remodeling balance is skewed toward increased resorption, this leads to osteoporosis. Such mechanisms underlying bone remodeling are explored through new research technologies such as genomics and proteomics. Deepening the understanding of the molecular mechanisms of bone remodeling has led to the development of various osteoporosis therapeutics that promote bone formation, inhibit bone resorption, or both.

In the treatment of osteoporosis, bisphosphonates combined with bone nutrients are the first-line treatments. The discovery of RANKL monoclonal antibodies and other novel drugs in the market shows how research into osteoporosis drug development is a fruitful area with further therapeutic potential. With the advancement of molecular biology and pharmacology, safer and more effective osteoporosis treatment options will continue to be identified and developed. We expect that clinical translational research employing new therapeutic methods such as stem cell therapy, miRNA inhibitors, and bone-targeting technology will bring breakthroughs in the treatment of osteoporosis.

## Data Availability

Not applicable.

## Abbreviations

AACE: American Association of Clinical Endocrinologists
ADSCs: Adipose-derived mesenchymal stem cells
PKB, Akt: Protein kinase B
AMOs: Anti-miRNA oligonucleotides
AP-1: Activator protein-1
BMD: Bone mineral density
BMPs: Bone morphogenetic proteins
BV/TV: Bone volume/total volume
Cbfa1: Core binding factor α 1
CXCR: Chemokine receptor
ERK1/2: Extracellular regulated protein kinases
ESCs: Embryonic stem cells
GFP: Green fluorescent protein
IL-1: Interleukin-1
iPSCs: Induced pluripotent stem cells
IκK: NF-κB inhibitor-κ-binding kinases
JNK: c-Jun N-terminal kinase
LGR4: Leucine-rich G-protein-coupled receptor 4
LRP: Low-density lipoprotein receptor-related protein
MALT1: Mucosa-associated lymphoid tissue lymphoma translocation factor 1
MAPK: Mitogen-activated protein kinases
M-CSF: Macrophage colony-stimulating factor
MMP: Matrix metalloproteinase
MSCs: Mesenchymal stem cells
NFATc1: Nuclear factor of activated T cells 1
NIK: NF-κB inducible kinase
NOD/SCID: Nonobese diabetic/severe combined immunodeficient
OPG: Osteoprotegerin
OPN: Osteopontin
OVX: Ovariectomized
PI3K: Phosphatidylinositol 3-kinase
PLGA/CoI: Polylactic acid polyglycolic acid copolymer/collagen type I
PP2A: Protein phosphatase 2A
PTEN: Gene of phosphate and tension homology deleted on chromosome 10
PTH: Parathyroid hormone
RANKL: Nuclear factor kappa B (NF-κB) ligand activated receptor
Runx2: Transcription factor runt-related transcription factor 2
SERMs: Selective estrogen receptor modulators
TB. N: Trabecular number
TB. Th: Trabecular thickness
TGF-β: Transforming growth factor-β
TNF-α: Tumor necrosis factor-α
TRAF-6: Tumor necrosis factor receptor-associated factor-6
UCB: Umbilical cord blood
VSELs: Very small embryonic-like cells
